# Supplementary material for: MetaRibo-Seq measures translation in microbiomes
Source: Nat Commun. 2020 Jun 29;11:3268. doi: 10.1038/s41467-020-17081-z (PMC7324362; doi:10.1038/s41467-020-17081-z)
Supplement: Supplementary file 10 — Supplementary Data 7 [file 41467_2020_17081_MOESM10_ESM.zip › File2/Confidence_High_Taxonomy/415349_out.krona.html]

Javascript must be enabled to view this page.

members
magnitude
magnitudeUnassigned
count
unassigned
taxon
rank

415349\_out

221

2
superkingdom
221

phylum
976
221

200643
class
221

order
171549
221

3
family
1853231

1
genus
574697

1
1472416
species

SRS053649\_contig\_number\_19952

283168
2

SRS019445\_contig\_number\_20686
1
genus

28118
species

SRS043701\_contig\_number\_10011
1

family
815
20

1
genus

SRS015431\_contig\_number\_44805
816
20

28116
species

SRS074964\_contig\_number\_3502SRS893256\_contig\_number\_547
2

818
species

SRS1041137\_contig\_number\_12095
1

3

SRS014412\_contig\_number\_9212SRS058723\_contig\_number\_1009SRS077335\_contig\_number\_1735
species
821

3
246787
species

SRS019267\_contig\_number\_3320SRS019685\_contig\_number\_37463SRS097920\_contig\_number\_1526

species
371601

SRS142503\_contig\_number\_39821
1

species
671267

SRS077127\_contig\_number\_13048
1

817
species

SRS075398\_contig\_number\_1696SRS075878\_contig\_number\_8654SRS140492\_contig\_number\_20710
3

1
species
457387

SRS053214\_contig\_number\_8889

3
46506
species

SRS020328\_contig\_number\_5271SRS050752\_contig\_number\_11770SRS146813\_contig\_number\_10586

1
species
469589

SRS045713\_contig\_number\_11935

171551
family
5

836
genus
5


SRS015190\_contig\_number\_16590SRS043411\_contig\_number\_11730SRS104975\_contig\_number\_13920SRS144297\_contig\_number\_8605SRS147346\_contig\_number\_17205
species
658663
5

193
family
2005525

193
375288
genus
46

SRS013098\_contig\_number\_27981SRS013638\_contig\_number\_contig-100\_655.100655SRS014313\_contig\_number\_641SRS016267\_contig\_number\_12534SRS017433\_contig\_number\_11339SRS017521\_contig\_number\_18313SRS018575\_contig\_number\_4839SRS019030\_contig\_number\_13410SRS019068\_contig\_number\_25397SRS019496\_contig\_number\_10583SRS019787\_contig\_number\_9056SRS019968\_contig\_number\_16484SRS021219\_contig\_number\_1751SRS023715\_contig\_number\_11237SRS024435\_contig\_number\_25869SRS043667\_contig\_number\_421SRS045244\_contig\_number\_4094SRS048060\_contig\_number\_3684SRS048164\_contig\_number\_contig-100\_1067.174178SRS048870\_contig\_number\_22244SRS052697\_contig\_number\_32742SRS056519\_contig\_number\_15604SRS063370\_contig\_number\_9109SRS075773\_contig\_number\_29889SRS075821\_contig\_number\_10764SRS077294\_contig\_number\_14083SRS077392\_contig\_number\_43417SRS077589\_contig\_number\_2954SRS097958\_contig\_number\_10913SRS098061\_contig\_number\_8308SRS098571\_contig\_number\_21496SRS098655\_contig\_number\_11936SRS1041039\_contig\_number\_20541SRS1041129\_contig\_number\_5943SRS1041147\_contig\_number\_1324SRS104693\_contig\_number\_8441SRS1055049\_contig\_number\_13044SRS1055069\_contig\_number\_4442SRS143085\_contig\_number\_2162SRS143876\_contig\_number\_25857SRS147271\_contig\_number\_23909SRS147653\_contig\_number\_873SRS148970\_contig\_number\_contig-100\_88.89SRS149075\_contig\_number\_contig-100\_1811.103947SRS149244\_contig\_number\_6964SRS971276\_contig\_number\_12336

4

SRS014235\_contig\_number\_27188SRS014287\_contig\_number\_9947SRS077086\_contig\_number\_3611SRS1055095\_contig\_number\_contig-100\_970.971
46503
species

species
574930

SRS020622\_contig\_number\_3385SRS046369\_contig\_number\_10716SRS098717\_contig\_number\_14118SRS1041090\_contig\_number\_900
4

species
328812

SRS971275\_contig\_number\_20771
1

107

SRS011084\_contig\_number\_15713SRS011134\_contig\_number\_15289SRS011239\_contig\_number\_10517SRS012273\_contig\_number\_41614SRS012969\_contig\_number\_31832SRS013800\_contig\_number\_11353SRS013951\_contig\_number\_30483SRS014459\_contig\_number\_27388SRS015065\_contig\_number\_25596SRS015217\_contig\_number\_18858SRS015579\_contig\_number\_13606SRS015663\_contig\_number\_13513SRS015782\_contig\_number\_2559SRS015794\_contig\_number\_9503SRS015890\_contig\_number\_13926SRS015960\_contig\_number\_11753SRS016056\_contig\_number\_186SRS016095\_contig\_number\_22630SRS016989\_contig\_number\_14352SRS017191\_contig\_number\_12733SRS017701\_contig\_number\_18719SRS018313\_contig\_number\_8667SRS018541\_contig\_number\_2563SRS018656\_contig\_number\_9988SRS018888\_contig\_number\_673SRS018984\_contig\_number\_4204SRS019161\_contig\_number\_31958SRS019178\_contig\_number\_524SRS019397\_contig\_number\_1042SRS020233\_contig\_number\_47015SRS021948\_contig\_number\_35294SRS022071\_contig\_number\_21274SRS022609\_contig\_number\_19188SRS023526\_contig\_number\_14933SRS024132\_contig\_number\_42177SRS024388\_contig\_number\_12719SRS044535\_contig\_number\_6507SRS045195\_contig\_number\_981SRS045826\_contig\_number\_14701SRS046712\_contig\_number\_145SRS049712\_contig\_number\_contig-100\_445.130219SRS049773\_contig\_number\_4386SRS049900\_contig\_number\_19623SRS050026\_contig\_number\_9813SRS050925\_contig\_number\_5808SRS050941\_contig\_number\_3677SRS051031\_contig\_number\_12041SRS051610\_contig\_number\_1658SRS051882\_contig\_number\_13832SRS052027\_contig\_number\_2602SRS052078\_contig\_number\_572SRS053398\_contig\_number\_10251SRS054590\_contig\_number\_609SRS056259\_contig\_number\_13282SRS057478\_contig\_number\_6067SRS058145\_contig\_number\_3805SRS058770\_contig\_number\_22482SRS062701\_contig\_number\_2252SRS063040\_contig\_number\_14777SRS064557\_contig\_number\_10172SRS064645\_contig\_number\_497SRS064973\_contig\_number\_508SRS065397\_contig\_number\_10295SRS065504\_contig\_number\_14141SRS076804\_contig\_number\_17234SRS076976\_contig\_number\_17548SRS077194\_contig\_number\_22644SRS077454\_contig\_number\_8845SRS077849\_contig\_number\_4691SRS078242\_contig\_number\_11991SRS078419\_contig\_number\_30527SRS078665\_contig\_number\_9315SRS100021\_contig\_number\_3082SRS103987\_contig\_number\_14355SRS104036\_contig\_number\_3492SRS1041036\_contig\_number\_567SRS1041091\_contig\_number\_16688SRS1041095\_contig\_number\_604SRS1041118\_contig\_number\_2895SRS1041132\_contig\_number\_2506SRS1041138\_contig\_number\_298SRS1041142\_contig\_number\_3287SRS104311\_contig\_number\_6167SRS1055038\_contig\_number\_790SRS142712\_contig\_number\_3194SRS143148\_contig\_number\_12635SRS143342\_contig\_number\_9880SRS143523\_contig\_number\_2271SRS144183\_contig\_number\_23868SRS144362\_contig\_number\_36653SRS144603\_contig\_number\_2714SRS145497\_contig\_number\_11999SRS147272\_contig\_number\_323SRS147377\_contig\_number\_5007SRS147614\_contig\_number\_1830SRS148511\_contig\_number\_15593SRS148784\_contig\_number\_15120SRS148874\_contig\_number\_2628SRS893187\_contig\_number\_11121SRS893231\_contig\_number\_244SRS893253\_contig\_number\_680SRS893259\_contig\_number\_463SRS893285\_contig\_number\_192SRS893288\_contig\_number\_126SRS893342\_contig\_number\_2188SRS893382\_contig\_number\_5761SRS893383\_contig\_number\_18719
823
species

2

SRS015264\_contig\_number\_7517SRS050299\_contig\_number\_2008
1947049
species

1

SRS150029\_contig\_number\_4816
species
2292053

6
species
469591

SRS017103\_contig\_number\_27108SRS018623\_contig\_number\_13744SRS022713\_contig\_number\_2852SRS023971\_contig\_number\_641SRS077730\_contig\_number\_607SRS1055099\_contig\_number\_14161

species
1262912

SRS018836\_contig\_number\_17628SRS019601\_contig\_number\_7963SRS020394\_contig\_number\_9215SRS023829\_contig\_number\_contig-100\_642.642SRS043768\_contig\_number\_29041SRS101268\_contig\_number\_contig-100\_1000.1001SRS1041134\_contig\_number\_6925SRS144506\_contig\_number\_23101SRS148196\_contig\_number\_39792
9

12
species
563193

SRS016335\_contig\_number\_33913SRS018351\_contig\_number\_17443SRS045645\_contig\_number\_24793SRS047014\_contig\_number\_35217SRS048981\_contig\_number\_58SRS056273\_contig\_number\_4108SRS064757\_contig\_number\_1749SRS075021\_contig\_number\_3271SRS075078\_contig\_number\_20991SRS1055076\_contig\_number\_1839SRS142890\_contig\_number\_22388SRS143722\_contig\_number\_14472

1
1750560
species

SRS893252\_contig\_number\_2385
